# Potential Links Between Physiological and Perceptual Strain in High Heat Stress

**DOI:** 10.1002/ejsc.70226

**Published:** 2026-09-09

**Authors:** Xiujing Zhao, Brendon P. McDermott, Wen‐Juo Lo

**Affiliations:** ^1^ University of Arkansas Fayetteville Arkansas USA; ^2^ College of Education and Health Professions University of Arkansas Fayetteville Arkansas USA

**Keywords:** heat strain index, perceived, sensation, subjective measures, thermoregulation

## Abstract

The physiological strain index (PhSI) is widely used to quantify thermoregulatory and cardiovascular strain during heat stress. However, direct physiological measurements may not always be feasible in occupational or athletic settings. Therefore, this study aimed to examine the relationship and agreement between perceptual strain and integrated physiological strain indices during high heat stress. Ten healthy, physically active, non‐heat‐acclimated males (29 (7) yr; 1.79 (0.11) m; 77.4 (9.3) kg) completed two randomized crossover exercise trials in hot‐dry (HD) and warm‐humid (WH) environments with equivalent wet‐bulb globe temperatures. Heart rate, rectal temperature, skin temperature, rating of perceived exertion, and thermal sensation were measured at baseline and every 15 minutes during 60 minutes of cycling. Physiological strain index (PhSI), adaptive physiological strain index (aPhSI), and perceptual strain index (PeSI) were calculated using validated equations. Repeated‐measures correlation analyses demonstrated very strong associations between PeSI and both PhSI and aPhSI under HD (*R*_rm_ = 0.940–0.943) and WH (*R*_rm_ = 0.980–0.982; all *p* < 0.001). Receiver operating characteristic analyses demonstrated good‐to‐excellent discrimination of physiological strain by PeSI (AUC = 0.895–0.969). Mixed‐effects analyses showed that higher PeSI values were associated with increased PhSI (*β* = 1.325, *p* < 0.001) and aPhSI (*β* = 1.459, *p* < 0.001). However, Bland‐Altman analyses demonstrated relatively small mean biases (0.4–0.9 AU) but wide limits of agreement (−2.8 to 4.2 AU), indicating that PeSI and physiological strain indices are not interchangeable. These findings suggest that PeSI may serve as a practical adjunctive or screening indicator of physiological strain when direct physiological measurements are unavailable.

## Highlights

- PeSI demonstrated very strong within‐subject associations with both PhSI and adaptive PhSI during moderate‐intensity cycling under hot‐dry and warm‐humid conditions.
- PeSI showed good‐to‐excellent discrimination of elevated physiological strain, with predictive performance consistently stronger in warm‐humid than hot‐dry conditions.
- Agreement analyses demonstrated wide limits of agreement, indicating that PeSI should not be considered interchangeable with physiological strain indices.
- PeSI may serve as a practical adjunctive screening tool for monitoring physiological strain when direct physiological measurements are unavailable

## 1 Introduction

Exercise or work in the heat challenges human physiological systems (e.g., thermoregulatory and cardiovascular) due to combined increases in metabolic heat production (e.g., muscle activity) and environmental heat load (Pokora and Żebrowska 2016). Heat stress impairs heat balance and results in an increase in heat strain. Notably, exposure to high heat stress challenges body thermoregulation by elevating body core temperature and increasing the risk of heat‐related illness (Borgetal. 2015; Cheung 2010; Lucas et al. 2014). Heat strain refers to physiological (e.g., thermal and cardiovascular) and perceptual responses to heat stress (Sawka et al. 2003). In this regard, the heart rate (HR), body core temperature (*T*_*c*_), and skin temperature (*T*_sk_) are commonly used indicators of physiological heat strain (Y. Yang and Chan 2015). As for perceptual heat strain, previous research proposed the perceptual strain index (PeSI) by attributing equal weight to the rating of perceived exertion (RPE) and thermal sensation (TS) to quantify the magnitude of perceptual strain during heat stress exposure (Tikuisis et al. 2002). Identifying the magnitude of heat strain is crucial to predict physiological and perceptual tolerance and protect people from heat‐related injury (Pokora and Żebrowska 2016).

Many heat strain indices were developed as holistic tools to determine the risk level of physiological or perceptual strains and predict tolerance times at various activities in a range of heat stress (Gagge et al. 1967; Lee 1980; Lee and Givoni 1971; Moran et al. 1998; Tikuisis et al. 2002). The physiological strain index (PhSI) was proposed by Moran et al. (1998) to track heat strain via thermoregulatory (*T*_*c*_) and cardiovascular (HR) strain. PhSI has been validated to assess heat strain across various climates (Montain et al. 1994), clothing ensemble, exercise intensities, hydration levels, ages, and sexes (Montain et al. 1994; Moran et al. 1998, 1999, 2002). Moreover, PhSI classifies heat strain as a single arbitrary value from 0 (no strain) to 10 (very high strain), which is helpful to quantify heat strain and easily compare varying environmental stressors (Moran et al. 1998; Y. Yang and Chan 2015). However, the calculation of PhSI relies on the direct, accurate measurement of *T*_*c*_ and HR. These physiological measures require sensitive devices directly attached to the individual; making this measure unsuitable for some field settings in extreme environments (e.g., firefighting and military operations) (Walker et al. 2017). In addition, considering necessary resources for large cohorts, the expense of measurement devices, and invasive measurement sites (e.g., rectal, esophageal), a simple and practical alternative monitoring protocol should be developed to overcome the limitations of PhSI.

Subjective perceptual measures such as RPE and TS may provide insight into physiological heat strain. RPE and TS have been used to complement physiological monitoring for athletes (G. Borg 1974; Young et al. 1987). Previous studies have demonstrated the close relation between perceptual measures of RPE and TS and physiological measures of *T*_*c*_ and HR (Aoyagi et al. 1998; Hostler et al. 2009; Marino et al. 2004; Vecellio et al. 2022). As such, modeled on PhSI, Tikuisis et al. (2002) proposed an integrated tool to assess heat strain, PeSI, which is calculated by attributing equal weight to RPE and TS. Their data suggests that the PeSI has the potential to estimate PhSI via RPE and TS (Tikuisis et al. 2002). In this regard, RPE reflects the subjective assessment of individual physical effort, which could act as a surrogate of HR. TS reflects thermal sensations arising from both peripheral and central thermal inputs and has been proposed as a perceptual surrogate of core temperature (Walker et al. 2017). In addition, PeSI is also a validated heat strain index that can identify heat strain in different fitness levels (Tikuisis et al. 2002), exercise intensities (Petruzzello et al. 2009), and clothing assembles (Petruzzello et al. 2009). Further, a plethora of investigations sought to determine whether PeSI can be an alternative to PhSI to assess heat strain at various heat stresses in laboratory or field settings (Borg et al. 2015; Borg et al. 2017; Chan and Yang 2016; Gallagher et al. 2012; Petruzzello et al. 2009; Tikuisis et al. 2002; Walker et al. 2017; Y. Yang and Chan 2015). Many studies revealed that PeSI was strongly associated with PhSI and can be a simple, robust, and user‐friendly tool for heat strain assessment in firefighting and occupational settings (Chan and Yang 2016; Petruzzello et al. 2009; Y. Yang and Chan 2015). Other studies suggest that PeSI can predict PhSI in varying heat stress [wet‐bulb global temperature (WBGT) of 21°C–37°C] in 50–120 minutes of low‐to‐moderate intensity exercise with a variety of protective garments (Borg et al. 2015; Borg et al. 2017; Gallagher et al. 2012). However, a study found that PeSI had a poor association with PhSI during both self‐paced and set‐paced firefighting tasks in the heat (Walker et al. 2017). Moreover, the fundamental mechanism of the PeSI in predicting PhSI is the ability of TS and RPE to provide an accurate subjective prediction of *T*_*c*_ and HR. Previous studies have reported that the relationship between TS and *T*_*c*_ (*r* = 0.28–0.72) is weaker than the relationship between RPE and HR (*r* = 0.81–0.92) (Borg et al. 2015; Borg et al. 2017; Casa et al. 2015; Gagge et al. 1967; Gallagher et al. 2012). Savage et al. (2014) also found that TS poorly predicted the *T*_*c*_. The responses to TS in the heat may be primarily driven by *T*_sk_ rather than *T*_*c*_ (Armstrong 2007; G. Borg 1970). Foster et al. (2021) reported that *T*_sk_ can adequately predict TS (*R* ^2^ = 0.840). Vecellio et al. (2022) also found that TS was associated with *T*_sk_, but not *T*_*c*_ during light physical activity in the heat. In consideration of the close relationship between TS and *T*_sk_, incorporating *T*_sk_ into the analysis may provide additional insight into the relationship between perceptual and physiological strain during varying environmental heat stress.

Although previous research has evaluated the effectiveness of PeSI for estimating physiological strain across diverse populations and conditions, including different environmental conditions (Borgetal. 2017), heat acclimation status (Janetos et al. 2026), occupational tasks (Chan and Yang 2016; Gallagher et al. 2012; Petruzzello et al. 2009; Y. Yang and Chan 2015), work rates (Borgetal. 2017; Janetos et al. 2026), clothing and protective equipment ensembles (Borg et al. 2015; Borg et al. 2017; Chan and Yang 2016; J. Yang et al. 2023; Y. Yang and Chan 2015), fluid temperature (O’Connor et al. 2025), age and health status (O’Connor, Meade, et al. 2024b), and sex (O’Connor, Meade, et al. 2024a), the relationship and agreement between perceptual and integrated physiological strain indices have not been evaluated in hot‐dry (HD) and warm‐humid (WH) environments with equivalent WBGT. Furthermore, incorporating skin temperature through the adaptive physiological strain index (aPhSI) may provide additional insight into the relationship between perceptual and physiological strain. Improved understanding of these relationships may facilitate the development of practical approaches for monitoring heat strain during work or exercise in hot environments. Therefore, the purpose of the present study was to examine the relationship and agreement between perceptual strain and integrated physiological strain indices (PhSI and aPhSI) during moderate‐intensity exercise in HD and WH environments with equivalent WBGT. As a secondary exploratory objective, we explored the relationships between PeSI and individual physiological responses (*T*_re_, HR, and *T*_sk_) to further characterize the physiological determinants of perceptual strain during high heat stress.

## 2 Methods

### 2.1 Participants

Ten healthy, physically active, and non‐heat‐acclimated males participated in this study (age: 29 (7) years; height: 1.79 (0.11) m; body mass: 77.4 (9.3) kg; body fat: 18.2 (5.6) %; body mass index: 23.9 (1.4); body surface area: 1.96 (0.18) m^2^; maximal oxygen consumption [$\dot{V}$O_2max_]: 51.9 (5.3) mL/kg/min). Participants were excluded if they self‐reported previous heat illness within the past 3 years, current musculoskeletal injury, or taking medications known to affect thermoregulation, or if they used tobacco or electronic cigarettes. Prior to testing, written informed consent was obtained from all participants before participation in accordance with the Declaration of Helsinki. All participants completed a medical history questionnaire prior to participation.

All experimental procedures were approved by The University Institutional Review Board prior to study data collection (IRB approved protocol number: 2309490663). All participants were asked to refrain from alcohol use for 24 hours, caffeine was matched for each trial, and strenuous activity was restricted for 24 hours before both preliminary and experimental trials. Participants wore a thin tee shirt or cycling jersey, shorts, socks, and cycling shoes that were matched between trials. Current data were collected as part of a large project, with main outcomes including thermoregulation and cardiovascular strain recently submitted elsewhere.

### 2.2 Environmental Conditions and Metabolic Rate

All trials were completed in an environmental chamber. A WBGT of approximately 30°C was achieved in both HD (39.2°C, 30% of relative humidity [RH]) and WH (34.2°C, 55% of RH) conditions. WBGT, ambient temperature, RH, and wind speed were measured every 15 minutes throughout (Kestrel 4400, Nielsen‐Kellerman, Boothwyn, PA, USA). The two randomized experimental trials (HD and WH) were separated by at least 6 days and occurred at the same time of day to account for circadian rhythm. Within each trial, participants completed 60 minutes of cycling at 55% of the wattage reached during the last stage of a preliminary $\dot{V}$O_2max_ test.

### 2.3 Preliminary and Experimental Protocol

During the preliminary visit, participants were familiarized with the environmental chamber, experimental protocols, and completed a $\dot{V}$O_2max_ test. Body composition was assessed via dual energy x‐ray absorptiometry prior to the experimental trial (DXA, Lunar Prodigy, General Electric, Madison, WI, USA). $\dot{V}$O_2max_ test was assessed via a cycle ergometer (Racer Mate, Seattle, WA) graded exercise protocol. Participants were informed on the RPE scale (G. Borg 1970), instrumented with 3‐lead ECG (Tango II, Suntech, Medical Inc., Morrisville, NC, USA), and adjusted the seat and handlebars prior to testing. Participants warmed up at 100W for 5 minutes at a self‐selected cadence. Following the warm‐up, resistance was increased by 25–50W every two minutes until the participant could not maintain 60 revolutions per minute on the cycle ergometer. During the $\dot{V}$O_2max_ test, HR, RPE, respiratory exchange ratio, and relative $\dot{V}$O_2_ were recorded every 30 seconds. The $\dot{V}$O_2_ value was considered maximal when at least two of the following criteria were met: (a) a plateau in oxygen consumption with increasing workload, (b) RER value > 1.15, and/or (c) HR ≥ 95% of age‐predicted HR maximum (HR_max_ = 220—age) and/or (d) RPE rating of 19–20 at the final stage.

During experimental trials, on arrival, participants provided a 24‐h urine sample to ensure euhydration, defined as urine specific gravity < 1.020 (USG, Master‐SUR, Atago Co Ltd, Tokyo, Japan) (Armstrong 2007). If USG exceeded 1.020, participants consumed 500 mL of water before commencing the trial. Participants then transitioned to the environmental chamber for a 10‐min upright seated acclimation period. After adjustment of the handlebars and seat, participants began a warm‐up for 2–5 minutes at a self‐selected load. Participants then began 60 minutes of cycling at 55% of the wattage reached during the last stage of the $\dot{V}$O_2max_ test. After 60 minutes of cycling, participants completed a 5‐min cooldown on the cycle ergometer at a self‐selected cadence and workload (matched between trials). After this, researchers removed instrumentation and measured nude body mass. Participants were provided refreshments, and future trials were scheduled, if necessary.

### 2.4 Physiological Outcomes Measure

HR was measured via a 3‐lead electrocardiogram (ECG) (Tango II, Suntech, Medical Inc., Morrisville, NC, USA). Rectal temperature (*T*_re_) was determined by a rectal thermistor (RET‐1, Physitemp Instrumental Inc., Clifton, NJ, USA) inserted ∼15 cm past the anal sphincter before participants started experimental trials. *T*_sk_ was continuously measured by thermocrons (iButton, Maxim Integrated, San Jose, CA, USA) attached with porous zinc oxide tape to the chest, triceps, thigh, and calf on the right side of the body to assess four‐site mean weighted skin temperature (Ramanathan 1964). *T*_re_ and *T*_sk_ data were continuously transmitted to a PowerLab data acquisition system and LabChart signal processing software (AD Instruments, Colorado Springs, CO). HR, *T*_re_, and *T*_sk_ were recorded at baseline and 15 minutes intervals, allowing the PhSI to be calculated at these intervals. These intervals were selected based on previous research (Borgetal. 2017; Tikuisis et al. 2002). The PhSI and the adaptive physiological strain index (aPhSI) were calculated using the following equations (Buller et al. 2023; Moran et al. 1998):

(1) $$PhSI=5\left[\left(\frac{T_{ret}-36.5}{39.5-36.5}\right)+\left(\frac{{HR}_{t}-60}{180-60}\right)\right]$$

(2) $$aPhSI=5\left[\left(\frac{T_{ret}-36.5}{\left(39.5+\frac{\left(T_{ret}-T_{skt})-4\right.}{4}\;\right)-36.5}\right)+\left(\frac{{HR}_{t}-60}{180-60}\right)\right]$$

*T*_ret_, HR_*t*_ and *T*_skt_ represent the *T*_re_, HR and *T*_sk_ at a particular time point. The PhSI and aPhSI attribute equal weight to thermoregulatory and cardiovascular measures, and rate physiological strain on a 0 to 10 scale. Strain was considered as: no/little (0–2.9), low–moderate (3–6.9), and high–very high (7–10) (Moran et al. 1998).

### 2.5 Perceptual Outcomes Measure

Perceptual parameters included RPE (G. Borg 1970) and TS (Toner et al. 1986) and were recorded every 15 minutes. RPE was measured using a previously validated 15‐point scale of 6 (very, very light) to 20 (very, very hard) (G. Borg 1970). RPE scales were visually presented to participants and accompanied by verbal instructions of “how hard are you working right now?” (G. Borg 1970). The participants responded by pointing to a number on a chart presented to them by researchers. Thermal sensation was measured using a 17‐point scale of 0.0 (unbearably cold) to 8.0 (unbearably hot) (Toner et al. 1986;AUT Walker et al. 2017). Similarly, participants were asked, “How hot or cold are you?” and responded by pointing to a number on a chart. We used the modified PeSI adapted by Petruzzello et al. (2009). To calculate, RPE and TS were transformed at each time point using:

(3) $$\text{PeSI}=5\left[\left(\frac{TS}{7}\right)+\left(\frac{RPE-6}{14}\right)\right]$$

Where TS and RPE represent values recorded at each measurement time point, similarly to the PhSI, strain was considered as: no/little (0–2.9), low–moderate (3–6.9), and high–very high (7–10).

### 2.6 Statistical Analysis

#### 2.6.1 Initial Analysis

Two‐way analysis of variance (ANOVA) with the factor of time (five levels: baseline, 15, 30, 45, and 60 minutes of cycling) and experimental condition (two levels: HD and WH) were used to compare differences in WBGT, *T*_re_, *T*_sk_, HR, PhSI, TS, RPE, aPhSI and PeSI between HD and WH conditions. Binary variables were compared with paired samples *t*‐tests. Repeated‐measures correlation analysis was conducted separately for each environmental condition to quantify within‐subject relationships between variables.

#### 2.6.2 Primary Analysis

Standard crosstab analysis with two‐level likelihood ratio calculations were conducted to assess whether PeSI could identify elevated physiological strain defined by PhSI ≥ 7 and aPhSI ≥ 7. The predictive power of the PeSI was evaluated by deriving four receiver operating characteristic (ROC) curves (Metz 1978), with the area under the curve (AUC) used to quantify discriminatory performance (perfect prediction = 1.0; random prediction = 0.5). A cut‐off of 7 was used to differentiate participants with high‐level physiological strain (Borg et al. 2015; Borg et al. 2017).

Mixed‐effects repeated measures analyses employing the subject‐specific maximum pseudo‐likelihood (MSPL) estimation method were conducted to explore how the PeSI predicts PhSI and aPhSI across five measurement points (from baseline to one hour at 15‐min intervals) under two environmental conditions. The model incorporated fixed effects for environmental conditions, time (in minutes), and the interactions between time and PeSI, along with a subject‐specific random intercept. An autoregressive [that is, AR (1)] correlation structure was employed to accommodate the serial correlation of repeated measurements within subjects. The MSPL method, a type of maximum pseudo‐likelihood estimation, was chosen due to its effectiveness in handling the hierarchical structure of the data and non‐normal distributions of random effects. This method is adept at addressing issues related to convergence and maintaining the positive definiteness of variance‐covariance matrices, challenges that are prevalent in analyses with small group sizes. This approach is particularly beneficial for the current dataset, characterized by constrained cluster sizes and variable group sizes, thus enhancing robustness in parameter estimation and reducing the occurrence of convergence issues and non‐positive definite *G* matrices commonly associated with traditional maximum likelihood methods. All data were recorded in Excel format. Data cleaning, format transformation, and analyses were completed using SAS 9.4 (SAS Institute Inc., 2023).

Agreement between PeSI and both physiological strain indices (PhSI and aPhSI) within each environmental condition was assessed using Bland‐Altman analyses to calculate the mean bias and 95% limits of agreement.

#### 2.6.3 Secondary Exploratory Analysis

As a secondary objective, we explored the relationships between PeSI and individual physiological responses. Mixed‐effects repeated measures analyses employing the subject‐specific maximum pseudo‐likelihood (MSPL) estimation method were conducted to explore how the PeSI predicts HR, *T*_sk_, and *T*_re_ across five measurement points (from baseline to one hour at 15‐min intervals) under two environmental conditions.

## 3 Results

### 3.1 Initial Analysis

**FIGURE 1:**
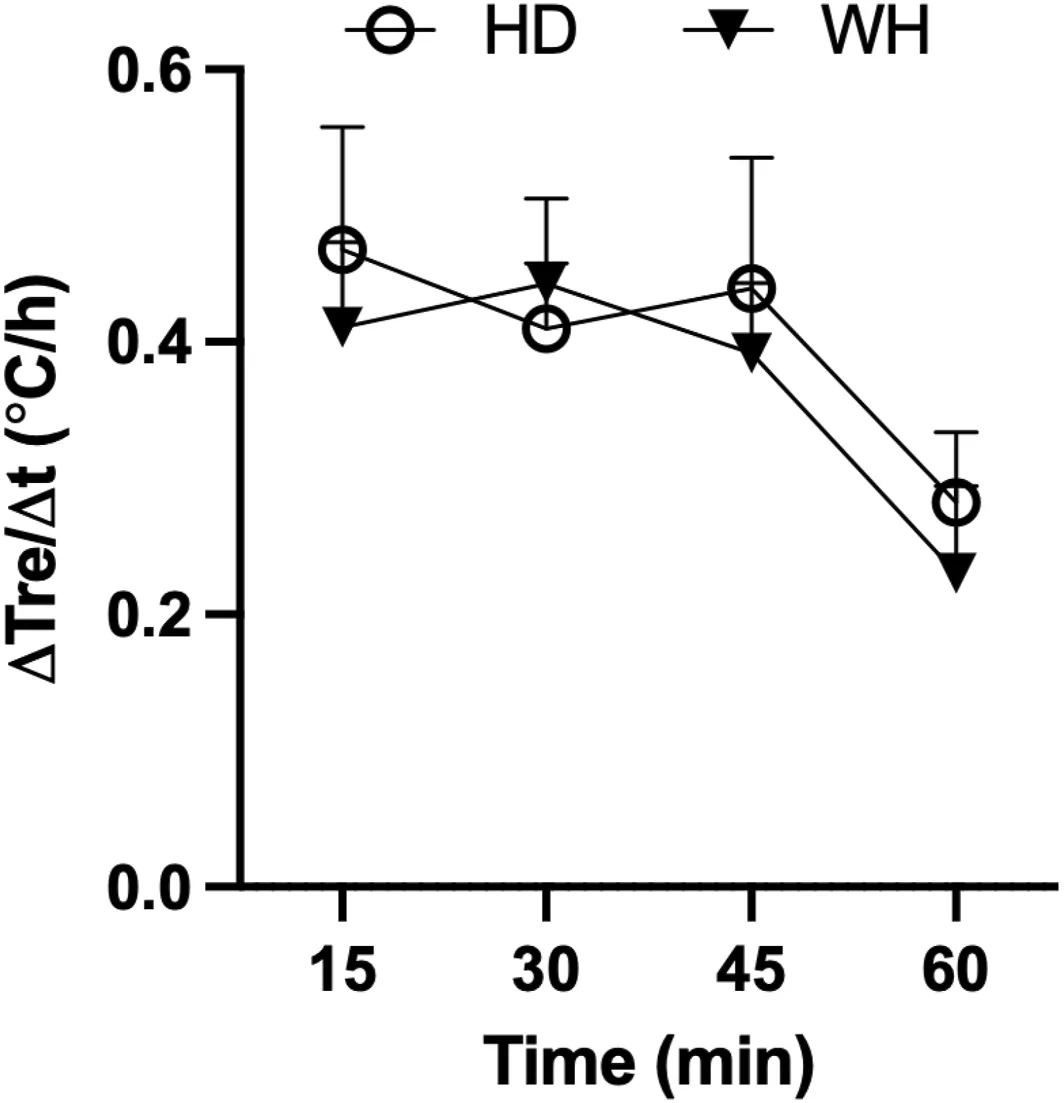
The rate of change in rectal temperature (Δ *T*_re_/Δt) during 60 minutes of cycling under hot‐dry (HD) and warm‐humid (WH) environmental conditions (*n* = 10).

**FIGURE 2:**
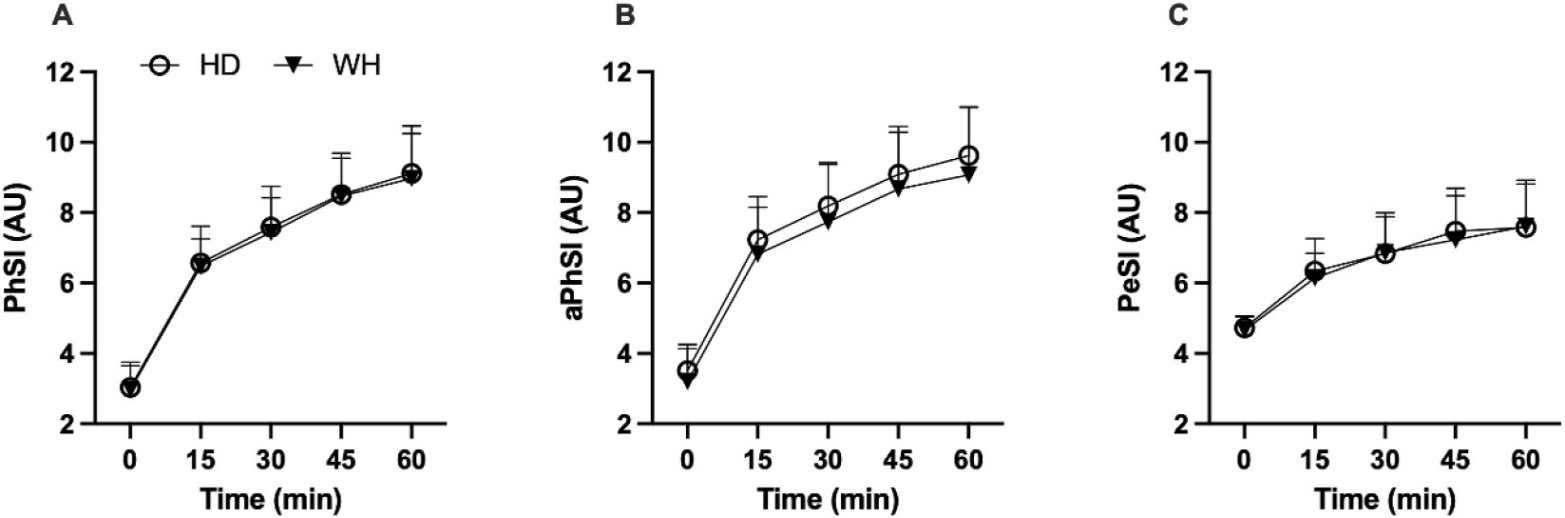
Changes in physiological strain index (PhSI; A), adaptive physiological strain index (aPhSI; B) and perceptual strain index (PeSI; C) during 60 minutes of cycling under hot‐dry (HD) and warm‐humid (WH) environmental conditions (*n* = 10).

Participants presented to the laboratory in a euhydrated state for both HD (USG: 1.011 [0.005]) and WH (USG: 1.012 [0.004]) condition trials. Figure 1 demonstrates that the rate of change in rectal temperature remained positive throughout the trials in both HD and WH conditions, indicating a progressive increase in rectal temperature during the high heat stress exposures. There were no significant differences in USG (*p* = 0.756), WBGT (*p* = 0.566), HR (*p* = 0.425), *T*_re_ (*p* = 0.687), RPE (*p* = 0.980), TS (*p* = 0.322), PhSI (*p* = 0.498), aPhSI (*p* = 0.498), or PeSI (*p* = 0.567) between HD and WH conditions. However, metabolic heat production (*p* = 0.043) and *T*_sk_ (*p* = 0.038) in HD were significantly greater than in WH conditions. Figure 2 presents changes in PhSI (Figure 2A), aPhSI (Figure 2B), and PeSI (Figure 2C) during 60 minutes of cycling under HD and WH environmental conditions.

**FIGURE 3:**
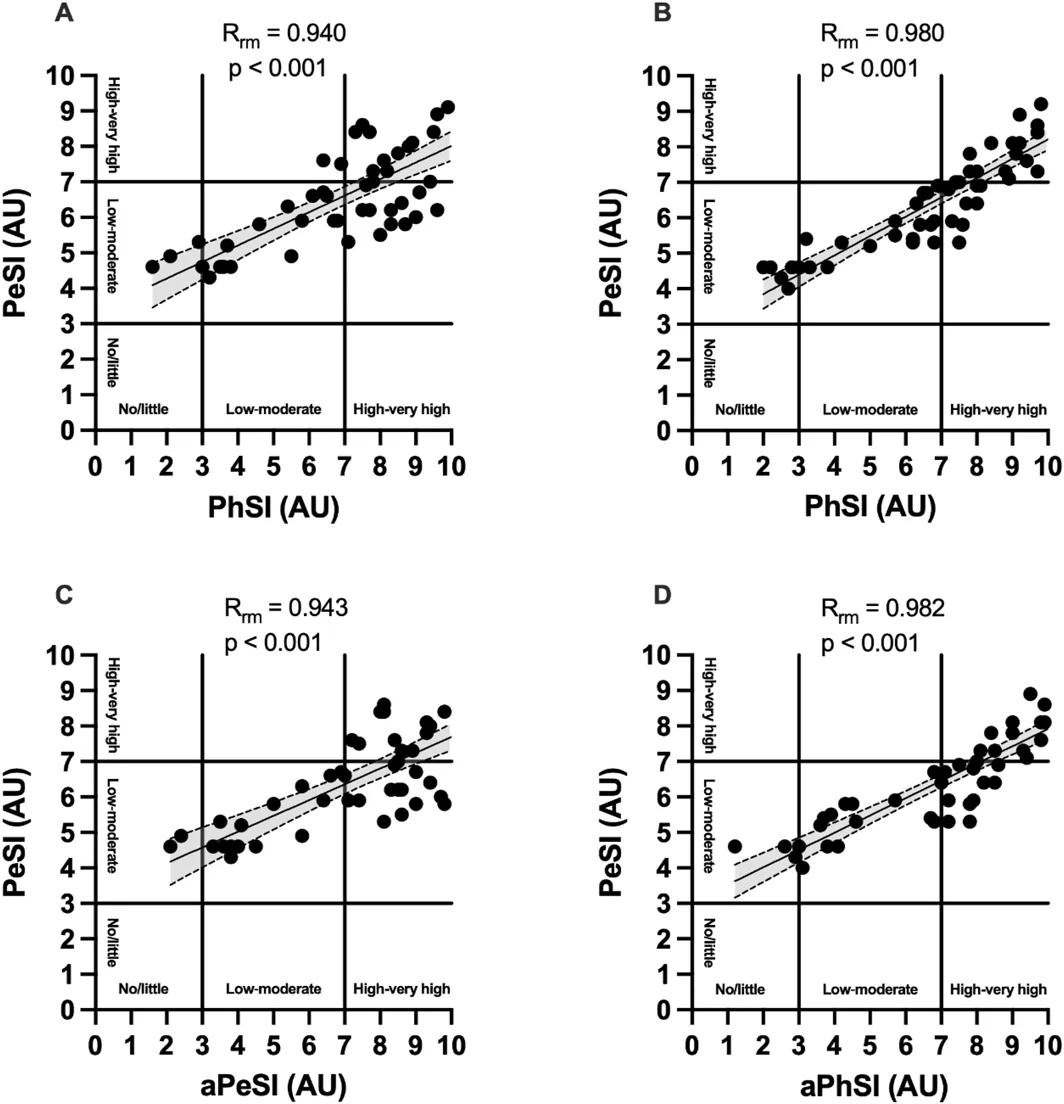
Repeated‐measures correlations between the perceptual strain index (PeSI) and the physiological strain index (PhSI; A: HD, B: WH) and the adaptive physiological strain index (aPhSI; C: HD, D: WH) under hot‐dry (HD) and warm‐humid (WH) environmental conditions. Vertical and horizontal lines denote the three strain index categories: no/little (0–2.9), low‐to‐moderate (3–6.9), and high‐to‐very high (7–10). Solid lines represent the repeated‐measures regression lines and shaded areas indicate the 95% confidence intervals.

Repeated‐measures correlation analyses demonstrated very strong positive associations between perceptual strain and both physiological strain indices under both environmental conditions (Figure 3). The association between PeSI and PhSI was very strong in the HD (*R*_rm_ = 0.940, 95% CI: 0.890–0.968, *p* < 0.001) and WH conditions (*R*_rm_ = 0.980, 95% CI: 0.963–0.990, *p* < 0.001). Similarly, PeSI was strongly associated with aPhSI in the HD (*R*_rm_ = 0.943, 95% CI: 0.894–0.969, *p* < 0.001) and WH conditions (*R*_rm_ = 0.982, 95% CI: 0.966–0.990, *p* < 0.001).

### 3.2 Primary Analysis

**FIGURE 4:**
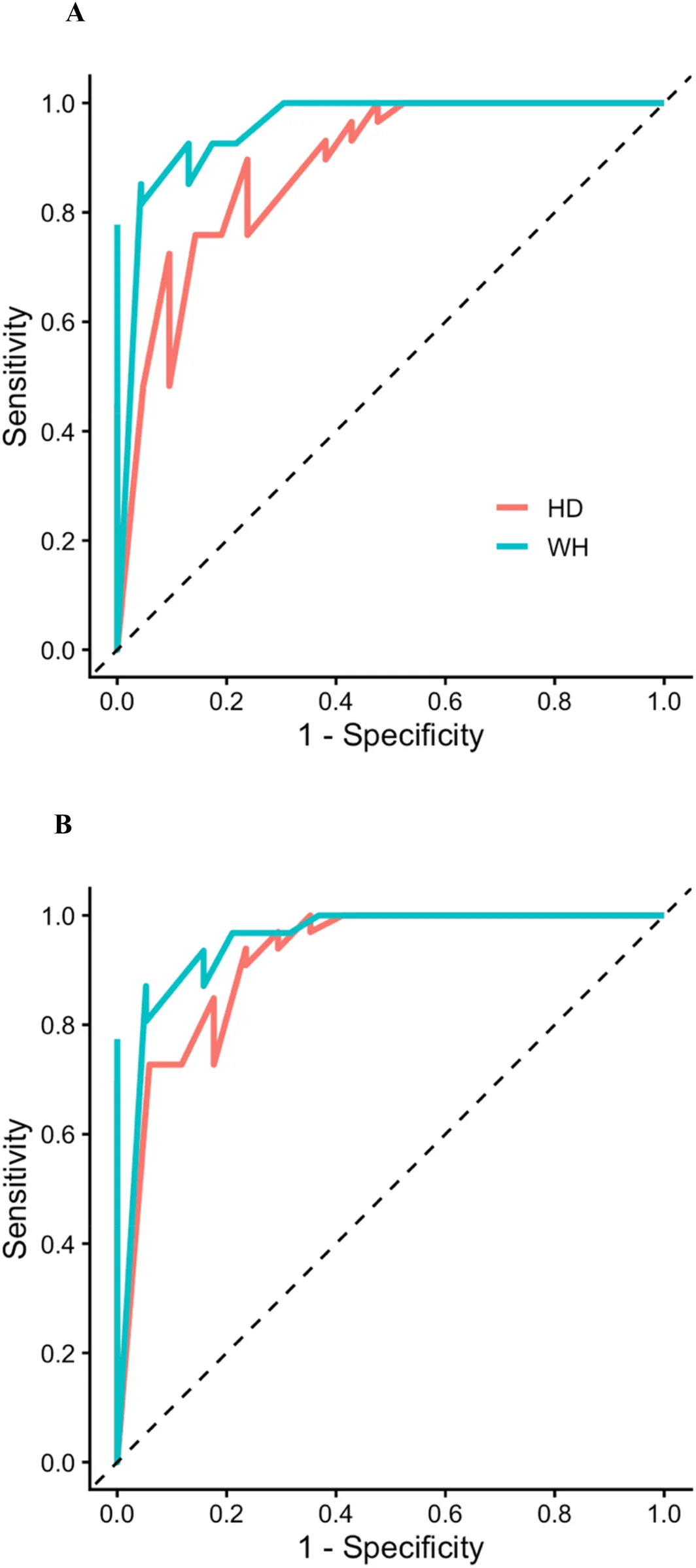
Receiver operating characteristic (ROC) curves for the perceptual strain index in identifying high physiological strain (≥ 7) based on (A) the physiological strain index (PhSI) and (B) the adaptive physiological strain index (aPhSI) under hot‐dry (HD) and warm‐humid (WH) conditions. The areas under the curve (AUC) for PhSI were 0.895 and 0.969 in the HD and WH conditions, respectively, whereas the AUCs for aPhSI were 0.939 and 0.969 in the HD and WH conditions, respectively. An AUC of 1.0 indicates perfect discrimination, whereas an AUC of 0.5 indicates discrimination no better than random chance.

Table 1 presents the results of the crosstab analyses, indicating that PeSI ≥ 7 significantly identified elevated physiological strain defined by PhSI ≥ 7 and aPhSI ≥ 7 under both HD and WH conditions (all Fisher's Exact Test *p* < 0.001). ROC analyses demonstrated good‐to‐excellent discrimination of high physiological strain by the PeSI (Figure 4). For identifying PhSI ≥ 7, the AUC was 0.895 (95% CI: 0.808–0.982) in HD and 0.969 (95% CI: 0.931–1.000) in WH. Similarly, for identifying aPhSI ≥ 7, the AUC was 0.939 (95% CI: 0.876–1.000) in HD and 0.969 (95% CI: 0.931–1.000) in WH.

**TABLE 1:** Predictive performance of perceptual strain index (PeSI) ≥ 7 for identifying physiological strain index (PhSI) ≥ 7 and adaptive physiological strain index (aPhSI) ≥ 7 in hot‐dry (HD) and warm‐humid (WH) conditions.

Condition	Case definition	TP	FP	FN	TN	Sensitivity (95% CI)	Specificity (95% CI)	LR+ (95% CI)	LR− (95% CI)
HD	PhSI ≥ 7	18	2	11	19	0.621 (0.440–0.773)	0.905 (0.711–0.973)	6.517 (1.692–25.106)	0.419 (0.258–0.681)
WH	PhSI ≥ 7	21	0	6	23	0.778 (0.592–0.894)	1.000 (0.857–1.000)	Not estimable^a^	0.222 (0.110–0.450)
HD	aPhSI ≥ 7	20	0	13	17	0.606 (0.437–0.753)	1.000 (0.816–1.000)	Not estimable^a^	0.394 (0.258–0.601)
WH	aPhSI ≥ 7	21	0	10	19	0.677 (0.501–0.814)	1.000 (0.832–1.000)	Not estimable^a^	0.323 (0.194–0.537)

Abbreviations: CI, confidence interval; FN, false negative; FP, false positive; TN, true negative; TP, true positive; LR+, positive likelihood ratio; LR−, negative likelihood ratio.

^a^ LR+ was not estimable because no false‐positive observations were observed.

Using PhSI as the dependent variable, significant main effects were observed for PeSI (*β* = 1.325, *p* < 0.001), indicating that higher levels of perceived strain were associated with increased physiological strain. Similarly, significant main effects of time on PhSI (*β* = 0.152, *p* < 0.001) suggest that physiological strain accumulated over time. Moreover, the significant PeSI × Time interaction (*β* = −0.016, *p* < 0.001) indicates that the association between PeSI and PhSI became weaker over time. The nonsignificant effects of environmental conditions on outcomes over time (*β* = 0.012, *p* = 0.921) suggest that PhSI remained similar under both HD and WH conditions. Fixed effect estimations for all models are detailed in Table 2.

**TABLE 2:** Fixed effect estimations (*n* = 10).

Model	*β*	SE	*p*
PhSI−model			
Condition	0.012	0.122	0.921
PeSI	1.325	0.110	< 0.001
Time	0.152	0.016	< 0.001
PeSI × time	−0.016	0.002	< 0.001
aPhSI−model			
Condition	0.192	0.137	0.200
PeSI	1.459	0.200	< 0.001
Time	0.170	0.018	< 0.001
PeSI × time	−0.019	0.003	< 0.001
Model−HR			
Condition	−0.248	2.258	0.915
PeSI	27.486	2.003	< 0.001
Time	3.209	0.284	< 0.001
PeSI × time	−0.427	0.042	< 0.001
Model−*T*_re_			
Condition	0.014	0.006	0.761
PeSI	0.104	0.040	0.011
Time	0.011	0.006	0.064
PeSI × time	0.001	0.0008	0.098
Model−*T*_sk_			
Condition	0.408	0.089	0.002
PeSI	0.273	0.077	< 0.001
Time	0.014	0.011	0.226
PeSI × time	−0.003	0.002	0.079

Abbreviations: aPhSI, adaptive physiological strain index; HR, heart rate; PeSI, perceptual strain index; PhSI, physiological strain index; *T*_re_, rectal temperature; *T*_sk_, skin temperature.

Using aPhSI as the dependent variable, a significant positive main effect of PeSI was observed (*β* = 1.459, *p* < 0.001), indicating that higher levels of perceived strain were associated with greater adjusted physiological strain. A significant positive main effect of time (*β* = 0.170, *p* < 0.001) further indicated that adjusted physiological strain increased over the course of the trial. Although the main effect of environmental condition was not significant (*β* = 0.192, *p* = 0.200), a significant negative interaction between PeSI and time (*β* = −0.019, *p* < 0.001) indicated that the association between perceptual strain and adjusted physiological strain became weaker as the trial progressed. Fixed‐effect estimates for all models are presented in Table 2.

**FIGURE 5:**
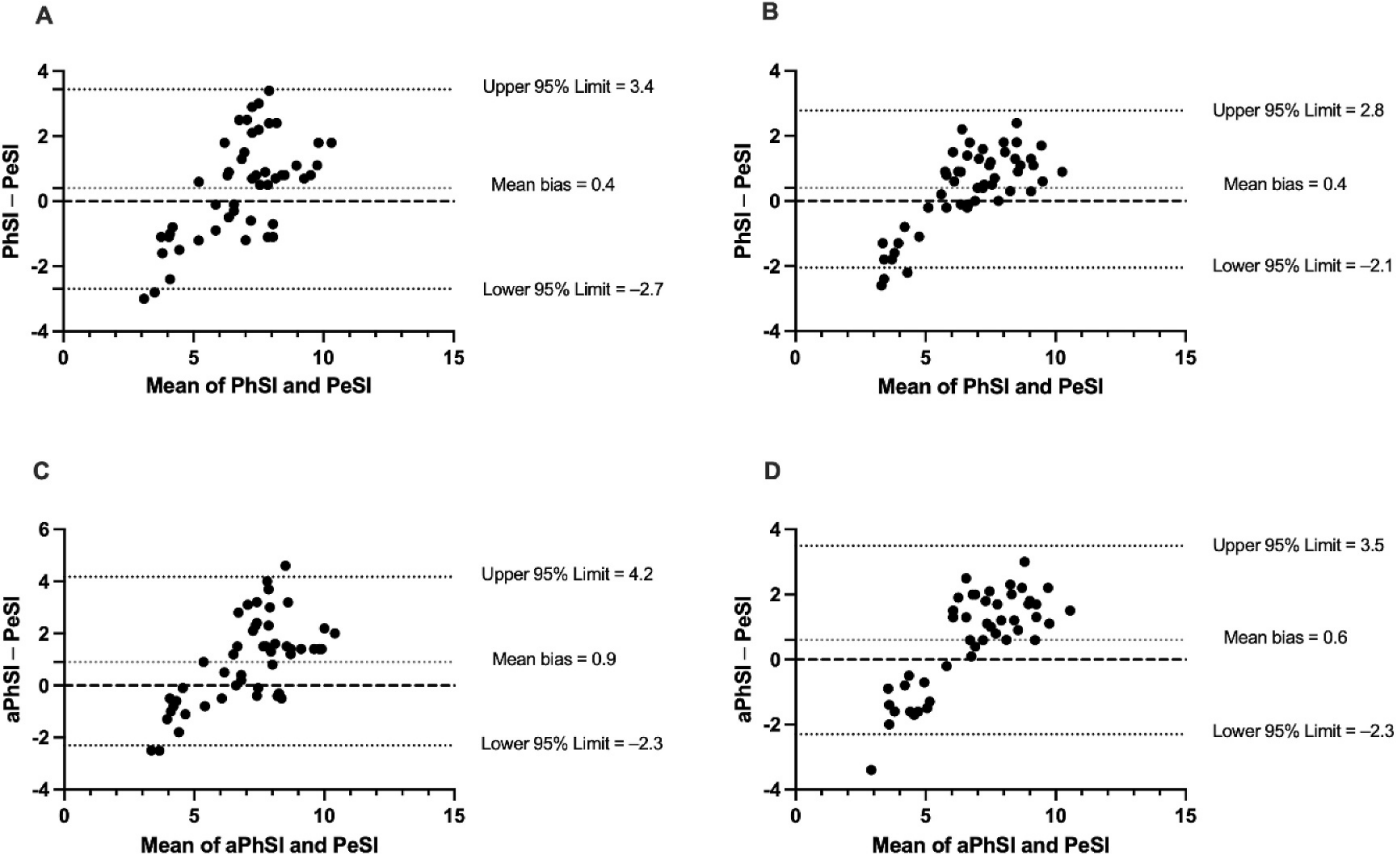
Bland–Altman plots illustrating the agreement between the perceptual strain index (PeSI) and the physiological strain index (PhSI; HD: A, WH: B) and between the perceptual strain index (PeSI) and the adaptive physiological strain index (aPhSI; HD: C, WH: D) under hot‐dry (HD) and warm‐humid (WH) conditions during moderate‐intensity cycling. The middle dashed line represents the mean bias, and the upper and lower dashed lines represent the 95% limits of agreement.

The mean bias between PeSI and PhSI did not differ significantly between HD (0.4 AU [95% LoA: −2.7 to 3.4], Figure 5A) and WH conditions (0.4 AU [95% LoA: −2.8 to 2.1], *p* = 0.949, Figure 5B). In contrast, the mean bias between PeSI and aPhSI was significantly greater in HD (0.9 AU [95% LoA: −2.3 to 4.2], Figure 5C) than in WH conditions (0.6 AU [95% LoA: −2.3 to 3.5], *p* = 0.018, Figure 5D).

### 3.3 Secondary Exploratory Analysis

Using HR as the dependent variable, significant main effects were observed for PeSI (*β* = 27.528, *p* < 0.001) and time (*β* = 3.208, *p* < 0.001), indicating that higher perceived strain and longer exercise duration were associated with higher HR. The PeSI × Time interaction was significant (*β* = −0.428, *p* < 0.001), indicating that the strength of the association between PeSI and HR decreased over time. Environmental condition was not significantly associated with HR (*β* = −0.252, *p* = 0.916).

Using Tre as the dependent variable, PeSI was positively associated with Tre (*β* = 0.103, *p* = 0.013). Neither time (*β* = 0.011, *p* = 0.067), the PeSI × Time interaction (*β* = 0.001, *p* = 0.109), nor environmental condition (*β* = 0.014, *p* = 0.765) were statistically significant.

Using Tsk as the dependent variable, significant positive associations were observed for PeSI (*β* = 0.274, *p* = 0.001) and environmental condition (*β* = 0.408, *p* = 0.002), indicating that higher perceived strain and environmental condition were associated with higher skin temperature. Neither time (*β* = 0.014, *p* = 0.238) nor the PeSI × Time interaction (*β* = −0.003, *p* = 0.086) were statistically significant.

## 4 Discussions

Although previous research suggests that PeSI can reflect physiological strain across diverse settings (Borg et al. 2015; Borg et al. 2017; Chan and Yang 2016; Janetos et al. 2026; O’Connor et al. 2024a, 2024b, 2025; Petruzzello et al. 2009; Walker et al. 2017; J. Yang et al. 2023; Y. Yang and Chan 2015), the present study is the first to evaluate the relationship and agreement between PeSI and both the PhSI and aPhSI in two distinct high heat stress environments (HD and WH) with equivalent WBGT during moderate‐intensity exercise. The primary findings were that: (1) very strong repeated‐measures associations were observed between PeSI and both physiological strain indices in HD and WH conditions; (2) higher levels of perceived strain were associated with greater physiological strain; (3) despite these strong relationships, Bland‐Altman analyses demonstrated relatively wide limits of agreement, indicating that perceptual and physiological strain indices should not be considered interchangeable measures; and (4) PeSI demonstrated good‐to‐excellent discrimination of physiological strain defined by PhSI ≥ 7 and aPhSI ≥ 7. Collectively, these findings suggest that PeSI may serve as a practical adjunctive or screening indicator of physiological strain in settings similar to the present experimental conditions, although additional validation under ecologically valid field conditions is warranted.

To more comprehensively understand the links between perceptual and physiological strain during heat stress exposure, the present study employed multiple analytical approaches. In agreement with previous investigations (Borg et al. 2015; Borg et al. 2017; Petruzzello et al. 2009; Walker et al. 2017), we observed very strong within‐subject associations between PeSI and both PhSI and aPhSI under both environmental conditions. Furthermore, significant positive relationships were observed between PeSI and individual physiological responses, including *T*_re_, HR, and *T*_sk,_ although the magnitude of these relationships varied by outcome and environmental condition. Interestingly, despite HD and WH conditions being matched for environmental heat stress (expressed by WBGT), the associations between PeSI and the physiological strain indices were stronger in WH than in HD conditions. This finding is consistent with the well‐established influence of environmental humidity and skin wettedness on thermal discomfort and physiological strain. High humidity reduces evaporative heat loss and increases skin wettedness, which may strengthen the coupling between perceptual and physiological responses (Buonocore et al. 2018; Fukazawa and Havenith 2009). However, the present analyses are observational and do not establish mechanistic causality. Moreover, the weak associations between PeSI and *T*_sk_ suggest that perceptual responses may be influenced by multiple peripheral and central thermal inputs rather than skin temperature alone. Collectively, these findings confirm and extend previous literature demonstrating relationships between perceptual and physiological strain into more severe heat stress conditions and moderate‐intensity exercise.

The mixed‐effects analyses further demonstrated that higher PeSI values were associated with increased PhSI, aPhSI, HR, *T*_re_, and *T*_sk_. Interestingly, although the mixed‐effects analyses did not identify a significant environmental condition effect, the correlation, agreement, and ROC analyses consistently suggested that PeSI performed better in the WH than HD condition. These findings suggest that perceptual responses generally track changes in physiological strain similarly across environments with equivalent WBGT despite differences in skin temperature responses.

However, correlation and predictive analyses alone may be inappropriate for evaluating whether PeSI can serve as a surrogate measure of physiological strain. Therefore, in line with previous investigations (Janetos et al. 2026; O’Connor et al. 2024a, 2024b, 2025; Walker et al. 2017; J. Yang et al. 2023), the present study employed Bland‐Altman analyses to quantify agreement between perceptual and physiological strain indices. Despite the very strong associations observed between PeSI and both PhSI and aPhSI, the agreement analyses demonstrated relatively wide limits of agreement, indicating substantial inter‐individual variability in the perception of physiological strain. Considering the 10‐point scale of the indices, these findings suggest that PeSI may substantially under‐ or overestimate physiological strain in some individuals, potentially resulting in the misclassification of physiological strain severity. Agreement between perceptual and physiological strain indices was generally better in the WH condition than in the HD condition, consistent with the stronger relationships observed under humid conditions. Therefore, although the mean biases were relatively small, the observed variability suggests that PeSI should not be considered interchangeable with either PhSI or aPhSI and is better interpreted as a practical adjunctive or screening indicator of physiological strain rather than a replacement for direct physiological monitoring.

Physiological strain is associated with reductions in physical work capacity and cognitive performance and an increased risk of heat‐related illness (Bouchama and Knochel 2002; Casa et al. 2015). Therefore, evaluating the ability of PeSI to identify elevated physiological strain when direct physiological measurements are unavailable has practical relevance. The present findings further demonstrate that PeSI exhibited good‐to‐excellent discrimination of elevated physiological strain defined by both PhSI ≥ 7 and aPhSI ≥ 7. Consistent with the relationship and agreement analyses, the predictive performance of PeSI was superior in the WH condition compared with the HD condition, suggesting that environmental characteristics, particularly humidity, may influence the utility of PeSI as a surrogate indicator of physiological strain. However, because the present study only examined two environmental extremes, the performance of PeSI under intermediate humidity conditions commonly encountered during occupational heat exposure remains unknown. These findings suggest that PeSI may help in identifying individuals experiencing elevated physiological strain in environments similar to those employed in the present study. Nevertheless, neither PhSI nor aPhSI represents a direct measure of unacceptable heat strain or heat‐related illness risk. Therefore, although PeSI demonstrated good discriminatory capability, its predictive performance is inherently limited by the strengths and limitations of these physiological strain indices as reference measures.

From a practical perspective, current occupational heat exposure limits are designed to protect most healthy, hydrated individuals from exceeding their capacity to maintain thermal equilibrium and are not based on physiological or perceptual thresholds. Nevertheless, perceptual measures may serve as useful complementary tools for individual heat strain monitoring when direct physiological measurements are impractical or unavailable. Therefore, PeSI may have utility as an adjunctive screening tool that can prompt increased surveillance or additional physiological monitoring rather than as a replacement for direct physiological assessment. Collectively, these findings provide preliminary evidence supporting the potential integration of perceptual measures into future heat monitoring strategies; however, additional validation in occupational, athletic, and military settings is required before operational implementation.

The current study should be interpreted in light of several limitations. First, the experimental protocol consisted of laboratory‐based moderate‐intensity cycling under tightly controlled environmental conditions. Therefore, the applicability of these findings to occupational, athletic, and military settings involving self‐pacing behaviors, variable workloads, solar radiation, protective clothing, and differing hydration practices should be interpreted cautiously. Future studies should evaluate the relationship between perceptual and physiological strain under ecologically valid field conditions. Second, the predictive models were developed from only 1 hour of moderate‐intensity exercise and may not be applicable to prolonged occupational exposures or lower‐intensity physical activities. Third, the relatively small sample size and the inclusion of only young, healthy, physically active males with high aerobic fitness limit the generalizability of the findings to females, older adults, and more representative occupational populations. Consequently, some predictive estimates should be interpreted cautiously and require validation in larger and more diverse cohorts. Fourth, the present study was not designed to determine heat compensability thresholds or identify an inflection point in core temperature. Therefore, the environmental conditions should be interpreted as high heat stress exposures rather than definitive evidence of uncompensable heat stress. Finally, although PeSI demonstrated strong relationships with physiological strain and good discriminatory capability, the relatively wide limits of agreement indicate substantial inter‐individual variability and suggest that PeSI should not be considered interchangeable with direct physiological measurements. Future studies should also evaluate the relationship and agreement between perceptual and physiological strain indices under varying clothing ensembles and prolonged heat exposure.

## 5 Perspectives

To our knowledge, this is the first study to evaluate the relationship and agreement between PeSI and both PhSI and aPhSI during moderate‐intensity exercise in HD and WH environments with equivalent WBGT. The present findings demonstrate that PeSI tracks changes in physiological strain and exhibits good‐to‐excellent discrimination of elevated physiological strain, with consistently better performance under WH conditions. However, the observed individual variability and imperfect agreement indicate that PeSI should not be considered a substitute for direct physiological monitoring. Instead, PeSI may serve as a practical adjunctive or screening tool when direct physiological measurements are unavailable or impractical. Additional studies involving larger and more diverse populations and ecologically valid field settings are needed before the operational implementation of PeSI for monitoring heat strain.

## Author Contributions

B.P.M.: supervision, conceptualized the study, writing – reviews and editing. X.Z.: conceptualized the study, writing – original draft. W. L., B.P.M., and X.Z.: analyzed the data. W.L.: writing – reviews and editing. All authors have read and agreed to the published version of the manuscript.

## Funding

The authors have nothing to report.

## Ethics Statement

Participation was voluntary and anonymous. Participants were free to withdraw at any time. All participants provided informed consent. The study protocol was approved by the University of Arkansas Institutional Review Board.

## Consent

Informed consent was obtained from all subjects involved in the study.

## Conflicts of Interest

The authors declare no conflicts of interest.

## Data Availability

The data present in this study are available on reasonable request from the corresponding author.
